# Text Matching in Insurance Question-Answering Community Based on an Integrated BiLSTM-TextCNN Model Fusing Multi-Feature

**DOI:** 10.3390/e25040639

**Published:** 2023-04-10

**Authors:** Zhaohui Li, Xueru Yang, Luli Zhou, Hongyu Jia, Wenli Li

**Affiliations:** 1 School of Maritime Economics and Management, Dalian Maritime University, Dalian 116026, China; 2 School of Economics and Management, Dalian University of Technology, Dalian 116024, China

**Keywords:** insurance question answering community, text matching, ERNIE, feature extraction, deep learning

## Abstract

Along with the explosion of ChatGPT, the artificial intelligence question-answering system has been pushed to a climax. Intelligent question-answering enables computers to simulate people’s behavior habits of understanding a corpus through machine learning, so as to answer questions in professional fields. How to obtain more accurate answers to personalized questions in professional fields is the core content of intelligent question-answering research. As one of the key technologies of intelligent question-answering, the accuracy of text matching is related to the development of the intelligent question-answering community. Aiming to solve the problem of polysemy of text, the Enhanced Representation through Knowledge Integration (ERNIE) model is used to obtain the word vector representation of text, which makes up for the lack of prior knowledge in the traditional word vector representation model. Additionally, there are also problems of homophones and polyphones in Chinese, so this paper introduces the phonetic character sequence of the text to distinguish them. In addition, aiming at the problem that there are many proper nouns in the insurance field that are difficult to identify, after conventional part-of-speech tagging, proper nouns are distinguished by especially defining their parts of speech. After the above three types of text-based semantic feature extensions, this paper also uses the Bi-directional Long Short-Term Memory (BiLSTM) and TextCNN models to extract the global features and local features of the text, respectively. It can obtain the feature representation of the text more comprehensively. Thus, the text matching model integrating BiLSTM and TextCNN fusing Multi-Feature (namely MFBT) is proposed for the insurance question-answering community. The MFBT model aims to solve the problems that affect the answer selection in the insurance question-answering community, such as proper nouns, nonstandard sentences and sparse features. Taking the question-and-answer data of the insurance library as the sample, the MFBT text-matching model is compared and evaluated with other models. The experimental results show that the MFBT text-matching model has higher evaluation index values, including accuracy, recall and F1, than other models. The model trained by historical search data can better help users in the insurance question-and-answer community obtain the answers they need and improve their satisfaction.

## 1. Introduction

Recently, an artificial intelligence question-answering system, ChatGPT, has attracted wide attention. ChatGPT mainly relies on natural language processing technology, and realizes the interaction of artificial intelligence through strong intention recognition and language understanding ability. Specifically, according to different application scenarios, the implementation difficulty of ChatGPT is also different. If ChatGPT is applied to information extraction, grammar correction and creative writing, the infrastructure provided by OpenAI can be directly applied to this. However, if it is applied to scenes that require high accuracy, such as medical consultation and business consultation, it is necessary to further improve the related technologies. In the future, the application scenarios of artificial intelligence will be continuously subdivided and applied to different vertical fields.

As one of the risk guarantees, with the steady rise of people’s living standards, people’s awareness of risks is increasing, and the demand for insurance is also increasing. Most users choose to obtain more information about the insurance industry through the Internet. Under this background, a question-and-answer community platform for the insurance field came into being. Compared with other comprehensive platforms, using natural language processing technology and professional insurance experience to dig deep into the vertical field can match the corresponding insurance problems more accurately and provide personalized services for users. At present, the rapidly increasing number of questions in the question-and-answering community and a large number of repeated, similar and irregular questions have brought great challenges to the traditional way of relying on manual customer service to answer questions of users. How to provide users with efficient, accurate and real-time question-and-answer services has become an urgent problem. Under the background of the current digital economy era, intelligent question-answering and intelligent recommendation through Internet technologies such as big data and text matching in text mining can effectively improve business processing efficiency, empower the economic development of the insurance industry, and achieve a win–win situation for insurance companies and users [1,2].

Text matching is one of the most fundamental and key technologies in applications such as intelligent question-answering systems and information retrieval [3], as well as an important research direction in the field of natural language processing. Aiming to improve the text-matching effect of the insurance library question-answering community, Deng et al. [4] integrated knowledge-based representation learning into the model by introducing an external knowledge base as a bridge, made use of cross-domain data to help complete tasks in the new domain, and proposed a cross-domain transfer learning framework for answer selection. Han et al. [5] weighted the keywords in the text and proposed a multi-granularity interaction method of context representation to achieve multi-granularity matching between questions and answers. Tan et al. [6] used a hybrid model of convolution and a recursive neural network to represent text features to improve the effect of question-and-answer matching. Andreas et al. [7] used a single BiLSTM for importance weighting in the text representation, and finally proved the effectiveness of this method. Bachrach et al. [8] enriched the feature representation of the text by combining the local information in a specific part of the answer with the global representation of the whole question and answer. Existing studies have proved that the performance of text matching can be improved by enriching the representation of text features, but there are still some problems such as the structure of the text feature extraction model being too simple, the mining of semantic features being insufficient, and the importance of proper nouns existing in insurance texts and the semantic ambiguity caused by polyphonic not being taken into account.

In this paper, aiming at problems such as too many proper nouns, poor sentence standardization and strong sparse features in question-and-answer text generated by the question-and-answer community in the insurance field, the semantic enhancement of the text is carried out from three aspects: word features, pinyin character features and part-of-speech features. At the same time, the influence of the global and local information of the text on the text-matching performance is considered. The MFBT model was constructed by using a pretraining language model, a cyclic neural network and a convolutional neural network, which improved the performance of text matching in the insurance field.

## 2. Related Work

### 2.1. Artificial Intelligence in the Insurance Field

Artificial Intelligence (AI), as the core driving force of insurance industry reform, is becoming increasingly prominent in its commercial value. The use of natural language processing in artificial intelligence technology not only solves the problem of human resource cost caused by high employee training costs and mobility, but also enhances user experience and satisfaction by tapping into user needs. At present, the research and application of artificial intelligence in the insurance field has achieved certain achievements, such as intelligent customer service, intelligent insurance consultants and intelligent question-and-answer systems [9].

How to understand the intention of the user more accurately and give quick answers according to the questions of users has become a hot research topic at present. Deng et al. [10] proposed that abstract extraction of answers could be used to generate more concise answers to solve the noise problem caused by too long answers, which is convenient for users to read and understand. Yuan et al. [11] proposed the use of deep learning to enhance the semantics and improve the accuracy of the task of answer selection by mining deeper semantics in a non-factual question-answering system. Bao et al. [12] proposed a double attention recurrent convolutional neural network, realized the interaction between questions and answers by using cross-attention, and conducted multidimensional semantic modeling of questions and answers, so as that the question-and-answer text can be better represented and improve the accuracy of the task of answer selection. Ha et al. [13] proposed that irrelevant information and non-standard expression in questions and answers should be paid attention to in community questions and answers, and the importance of redundant and noisy texts should be reduced through an attention mechanism, while the expression of important fragments should be emphasized, so as to better select correct answers. Mozafari et al. [14] demonstrated that the interaction between questions and answers can effectively improve the accuracy of answer selection. Zhang et al. [15] used an attention mechanism to measure the importance of each fragment in a text, and differentiated texts to focus on those fragments that are more useful for answer selection. Jing et al. [16] proposed that the performance of text matching in the question-and-answering community can be improved by introducing the expertise and authority of respondents for knowledge enhancement. In addition, for the question-and-answering community in vertical fields, professional knowledge in vertical fields can be effectively obtained by integrating external knowledge graphs, thus improving the performance of answer selection in the question-and-answering community. Jagvaral et al. [17] acquired the semantic correlation between entities by integrating a convolutional neural network, BiLSTM and an attention mechanism, and combined with knowledge graph technology, predicted the relationship between entities and candidate sets through paths.

The intelligent question-answering system in the insurance field excavates the deep-seated needs of users through natural language processing technology to enhance the user experience, aiming at helping users understand and choose insurance products and improving their risk prevention ability. On the basis of previous studies, combined with relevant professional knowledge and data characteristics in the field of insurance, this paper improves the answer selection performance of the insurance question-answering community through multi-feature representation and the introduction of prior knowledge.

### 2.2. Text Matching

Text matching is a basic task in natural language processing. Many tasks of artificial intelligence in the insurance field can be described as text-matching problems, such as questions and answers about insurance knowledge and the answer selection in the insurance field. Among them, the task of knowledge questions and answers is to find similar questions according to the query and then return the answers, while the task of answer selection is to select the most suitable one from the candidate answers according to the questions input by users. In recent years, because deep knowledge has powerful representational and cognitive functions, many researchers began to apply it to complex text-matching problems. At present, the application of deep learning model in text matching can be mainly divided into three types: single-semantic text representation, multi-semantic text representation and text representation of direct modeling matching patterns [18].

The deep model based on single-semantic text expression uses Convolutional Neural Networks (CNNs) [19,20], Recurrent Neural Networks (RNNs) [21,22] and other deep learning models to express the two documents that must be matched into two high-dimensional dense vectors, and use the twin network architecture [23] to judge whether the two documents are matched by calculating the similarity of the two vectors. Because of its coding independence, the single-semantic document representation model can calculate and store the text vectors in advance, thus improving the matching speed. However, the single-semantic model does not consider the local structure information of the text, and cannot process long sequence text.

In order to solve the problem of information loss in learning text features with the single-semantic model, researchers proposed a depth model of multi-semantic document representation that is more suitable for processing long-sequence texts based on the single-semantic document representation model. By comprehensively considering the local information such as words and phrases, and the global information such as sentences and paragraphs, this model realizes the multi-granularity feature representation of the text [24,25,26,27].

Document representation models based on single-semantics and multi-semantics focus on how to better represent a single text to a vector, ignoring the interaction between text and text. Different from the above two models, the direct modeling matching pattern model interacts two text pairs to be matched in the input stage, which effectively solves the problem of matching information loss caused by the abstract text expression on the basis of retaining the word granularity matching information. However, the direct modeling matching pattern cannot calculate and store the vector information of the text in advance, so it has the disadvantage of time consumption. The MFBT model proposed in this paper is based on multi-semantic representation documents and direct modeling matching patterns.

## 3. Construction of Text Matching Model

**Figure 1:**
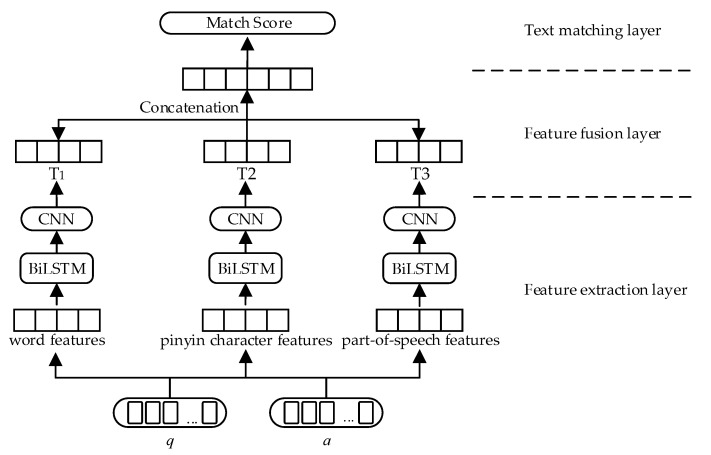
Framework of the MFBT model.

In order to make the text matching model more suitable for the insurance question-and-answer community dataset, this paper proposes the MFBT text matching model, which integrates BiLSTM and TextCNN fusing multi-feature, and its frame diagram is shown in Figure 1, where q represents the question and a represents the answer.

The MFBT model consists of three parts: a feature extraction layer, a feature fusion layer and a text matching layer. After preprocessing operations such as word segmentation and denoising, the preprocessed text first goes through the semantic expansion stage of the feature extraction layer, which realizes semantic enhancement by extracting the word features, pinyin character features and part-of-speech features of the text. Then, the expanded text feature representation is taken as the input of the BiLSTM and TextCNN, and the representation of the global and local features of the text is obtained successively. The extracted features are then input into the feature fusion layer, which fuses the extracted features to obtain the final text feature representation. Finally, the text matching layer is used to measure whether the question matches the candidate answer.

### 3.1. Problem Definition

This paper mainly studies the text-matching problem in a question-and-answer retrieval system in the field of insurance. During training, a set of triples $\left\{\left(d_{q},d_{a},\;x\right)\right\}$ is given, where $d_{q}$ is the search term, $d_{a}$ is the sample data, and $x\in \left\{0,\;1\right\}$ is the label data. When x=1, it means that $d_{q}$ matches $d_{a}$; on the contrary, when x=0, it means that $d_{q}$ and $d_{a}$ do not match.

The goal of question matching is to determine whether $d_{q}$ matches $d_{a}$, in which case they are given a set $\left\{\left(d_{q},d_{a}\right)\right\}$.

### 3.2. Feature Extraction Layer

The MFBT model extracts text features from five aspects: word features, pinyin character features, part-of-speech features and global and local semantic features. This section mainly introduces the specific feature information of these five aspects and the corresponding extraction methods.

#### 3.2.1. Word Features Extraction

**Figure 2:**
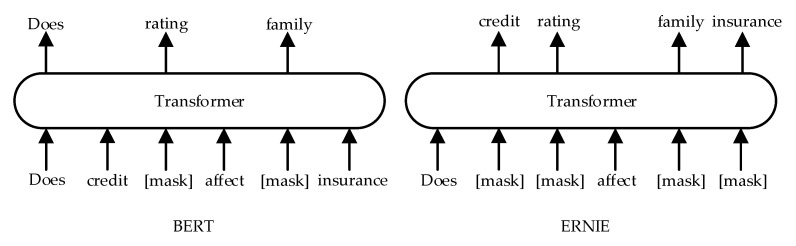
The different masking strategy between BERT and ERNIE.

Word feature is the sequence representation of phrases, which is one of the basic features of the text. In the stage of word feature extraction, this paper uses the ERNIE model [28] to obtain the word vector representation of the original text. Baidu proposed the ERNIE model based on the BERT (Bidirectional Encoder Representation from Transformers) model [29]. Compared with BERT, ERNIE mainly improves the MASK mechanism. The MASK stage of ERNIE includes basic-level masking, entity-level masking and phrase-level masking. In the pre-training stage, BERT first covers 15% of the words randomly, and then predicts the covered words. Although this method greatly improves the prediction performance, it ignores the relationship between word and word, whereas the MASK mechanism of ERNIE predicts the whole by covering phrases, named entities, etc. The semantic information of proper names in the insurance field is preserved to the maximum extent. ERNIE in the MFBT model proposed in this paper is only used to acquire the word vector representation of the text. The different masking strategy between BERT and ERNIE is shown in Figure 2.

Using ERNIE for word vector acquisition not only considers the long-distance dependent information of the text, but also solves the problem that BERT and other models lack prior knowledge when carrying out the text representation. Text $s=\left\{w_{1},w_{2},\ldots ,w_{n}\right\}$ after word segmentation, where n represents the number of words after text segmentation, and $w_{i}$ represents the i word. First, ERNIE can obtain a text representation with rich semantic information by integrating three different MASK strategies, and then input it into Transformer to generate a word vector sequence. Among them, the hidden layer of ERNIE consists of 768 dimensions and the sequence length consists of 128. Finally, the word embedding vector of the text is obtained by training the embedding layer of the ERNIE model. Vector $v_{i}^{w}$ is obtained after text word vector training. The calculation process is shown in Formula (1), where $V_{1}$ is the vectorization process of ERNIE. The text $E_{1}$ after vectorization is taken as an input of the model, and its construction formula process is shown in Formula (2).

(1) $$v_{i}^{w}=V_{1}\left(w_{i}\right)$$

(2) $$E_{1}=\left[v_{i}^{w}\right]$$

#### 3.2.2. Pinyin Character Features Extraction

Chinese pinyin is a tool to assist the pronunciation of Chinese characters. In Chinese, the same Chinese character may have different pinyin, and different pinyin represent different meanings. In order to solve the problem that homophones and polyphones in the text affect the representation of text features, this paper introduces the pinyin characters of Chinese characters to distinguish between them. For example, “[What does car insurance cover]” can be expressed as “[qichebaoxianhangaishenme]” in Pinyin. The text features are enhanced by combining the characteristics of Chinese characters and pinyin. The text is represented by a sequence of pinyin characters, and then the characters are embedded. Specifically, in the pre-processing stage, the text is transformed into a single character, and then the pinyin of the single character is obtained. Then, the pinyin of a single character is matched with the dictionary meaning established for the corresponding text. Finally, the word embedding vector set $T_{k}$ is obtained after large-scale corpus training. Then, it is necessary to vectorize the pinyin character sequence to obtain the pinyin character vector $v_{i}^{c}$ of the text. Finally, the character vector representation of the text is acquired by establishing the index between pinyin and Chinese characters. In this paper, the vector dimension of pinyin character features is set to 100 dimensions. The vectorization calculation process of pinyin characters is shown in Formula (3), where $V_{2}$ is the one-hot vectorization process, and after vectorization the text feature $E_{2}$ is taken as an input of the model, and its construction process is shown in Formula (4).

(3) $$v_{i}^{c}=V_{2}\left(w_{i}\right)$$

(4) $$E_{2}=\left[v_{i}^{c}\right]$$

#### 3.2.3. Part-of-Speech Features Extraction

As the basic attribute of words, different parts of speech are of different importance in the text. In order to acquire deeper semantic features of the text, this paper extracts the parts of speech of the text as features. The data after word segmentation is marked in accordance with the part of speech tagging specification of Peking University [30]. Apart from the conventional marking of nouns, verbs, adjectives, etc., the part of speech is also specially defined for proper nouns in the insurance field, which is more convenient for model recognition and learning of text features in the insurance professional field. The marked part of speech is used one-hot for part of speech feature vector quantization. For the convenience of calculation, this paper sets the vector dimension of part-of-speech features to 50 dimensions. The part of speech feature $v_{i}^{p}$ is obtained, and its vectorization process is shown in the Formula (5), where $V_{3}$ is the one-hot vectorization process. Since the parts of speech cannot exist separately from the words, this paper combines the acquired part-of-speech features with the word features to obtain the input $E_{3}$ of the model. The construction process is shown in Formula (6).

(5) $$v_{i}^{p}=V_{3}\left(w_{i}\right)$$

(6) $$E_{3}=\left[v_{i}^{w},v_{i}^{p}\right]$$

#### 3.2.4. Global Feature Extraction

Global feature extraction is the acquisition of the text context information. Most question–answer corpora in the insurance community are long text sequences. In order to better capture the long-distance dependent information in the corpora, this paper uses the BiLSTM model to extract global features from the input vectors $E_{1}$, $E_{2}$ and $E_{3}$.

**Figure 3:**
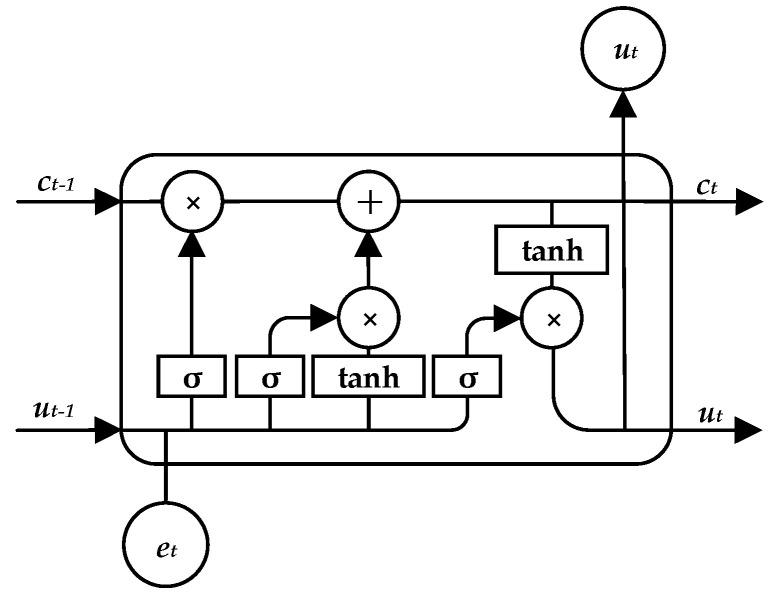
Schematic of LSTM.

Long Short-Term Memory (LSTM) is a special case of cyclic neural networks. Compared with the original RNN structure, LSTM adds a forgetting gate, input gate and output gate in each unit. It not only solves the problem of reverse gradient disappearance existing in traditional RNN, but also is more suitable for modeling long text data. The structure of LSTM [31] is shown in Figure 3.

Figure 3 shows the computational state inside LSTM neurons at time t. Each neuron consists of three inputs and three outputs, where $e_{t}$ is the newly added information at the time of t, namely the input vector of the model; and $c_{t-1}$ and $u_{t-1}$ are respectively the state of the neuron at the last moment and the output value of the neuron at the last moment, which is the representation of the above information. With forget gates and input gates, LSTM can effectively retain useful information in text sequences. However, a one-way LSTM can only model text in one direction, and cannot acquire text context information at the same time.

**Figure 4:**
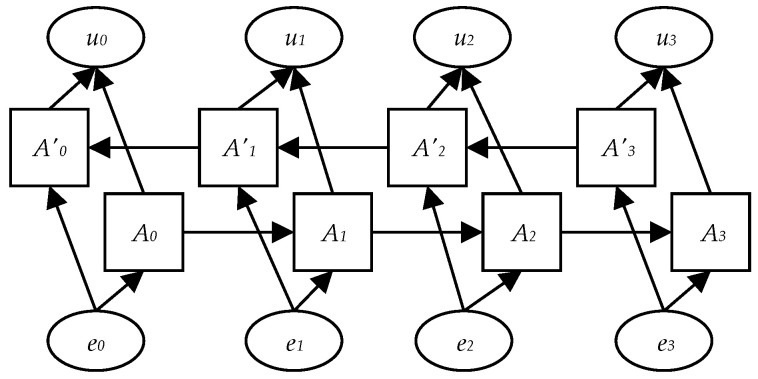
Schematic of BiLSTM.

In view of the problems existing in unidirectional LSTM, Graves et al. [32] added the reverse operation on the basis of unidirectional LSTM and proposed the BiLSTM model, which includes forward LSTM and reverse LSTM. By modeling the text in both directions, contextual information associated with words can be mined more comprehensively. The structure of BiLSTM [32] is shown in Figure 4.

(7) $$u_{i}^{d_{q}}=\mathrm{BiLSTM}\left(e_{w_{d_{q}}i},E_{j}\right)\in R^{1\times 2k_{1}},\forall i\in \left[0,\cdots ,N_{d_{q}}\right]\;$$

(8) $$u_{i}^{d_{a}}=\mathrm{BiLSTM}\left(e_{w_{d_{a}}i},E_{j}\right)\in R^{1\times 2k_{1}},\forall i\in \left[0,\cdots ,N_{d_{a}}\right]\;$$

where $E_{j}$ is the input of semantic features, $j\in \left(1,2,3\right)$, $k_{1}$ represents the number of hidden layers of the BiLSTM model, and $N_{d_{q}}$ and $N_{d_{a}}$ respectively represent the number of tokens contained in the input text $d_{q}$ and $d_{a}$.

Therefore, through the BiLSTM model, we can obtain the global features $U_{d_{q}}$ and $U_{d_{a}}$ of the text, which contains the context information, as shown in Formulas (9) and (10).

(9) $$U_{d_{q}}=\left[u_{1}^{d_{q}};\cdots ;u_{N_{d_{q}}}^{d_{q}}\right]\in R^{N_{d_{q}}\times 2k_{1}}\;$$

(10) $$U_{d_{a}}=\left[u_{1}^{d_{a}};\cdots ;u_{N_{d_{a}}}^{d_{a}}\right]\in R^{N_{d_{a}}\times 2k_{1}}\;$$

#### 3.2.5. Local Feature Extraction

On the basis of global feature extraction, this paper also considers the local features of the text, and uses the TextCNN model to extract the local features of the text.

**Figure 5:**
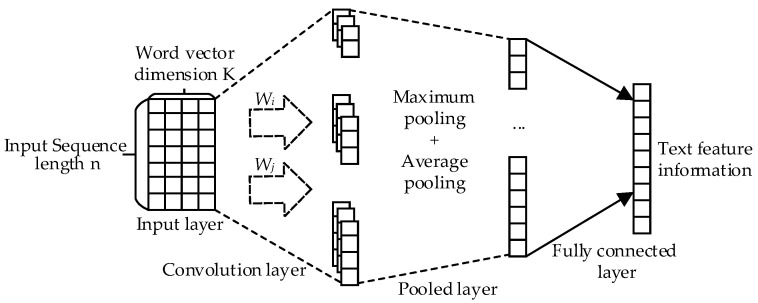
Schematic of TextCNN.

TextCNN is an application of CNN proposed in the field of natural language processing on the basis of CNN. Due to its features such as a simple structure, a strong local feature extraction ability and fast speed, TextCNN is widely used in the field of natural language processing. The network architecture of TextCNN [20] is shown in Figure 5.

In Figure 5, the input of TextCNN is $U_{d_{q}}$ and $U_{d_{a}}$ obtained after the text extracted by BiLSTM, and the obtained feature vector of the text matrix after the TextCNN model is:

(11) $$C_{d_{q}}^{i}=\mathrm{TextCNN}\left(d_{q},E_{j},f_{i}\right)\in R^{1\times N_{d_{q},f_{i}}}\;$$

(12) $$C_{d_{a}}^{i}=\mathrm{TextCNN}\left(d_{a},E_{j},f_{i}\right)\in R^{1\times N_{d_{a},f_{i}}}\;$$

where i represents which convolution kernel it goes through.

In this paper, the method of maximum pooling plus average pooling is used. The feature vectors $C_{d_{q}}^{i}$ and $C_{d_{a}}^{i}$ are passed through the pooling layer to reduce the number of parameters in the connection layer and prevent overfitting. The average pooling process is shown in Formulas (13) and (14):

(13) $$u_{avg}^{d_{q},i}=\frac{1}{N_{d_{q},f_{i}}}\sum_{i=0}^{N_{d_{q},f_{i}}}C_{d_{q}}^{i}\;$$

(14) $$u_{avg}^{d_{a},i}=\frac{1}{N_{d_{a},f_{i}}}\sum_{i=0}^{N_{d_{a},f_{i}}}C_{d_{a}}^{i}$$

The maximum pooling process is shown in Formulas (15) and (16):

(15) $$u_{max}^{d_{q,i}}=\underset{0⩽i⩽N_{d_{q,},f_{i}}}{max}C_{d_{q}}^{i}$$

(16) $$u_{max}^{d_{a,i}}=\underset{0⩽i⩽N_{d_{a,f_{i}}}}{max}C_{d_{a}}^{i}$$

After the text matrixes $U_{d_{q}}$ and $U_{d_{a}}$ pass through the pooling layer, the average pooling results $U_{avg}^{d_{q}}$ and $U_{avg}^{d_{a}}$ and the maximum pooling results $U_{max}^{d_{q}}$ and $U_{max}^{d_{a}}$ are obtained, and then the results are spliced. The splicing process is shown in Formulas (17) and (18), where ⊕ is the splicing process.

(17) $$W_{d_{q}}=U_{avg}^{d_{q}}⊕U_{max}^{d_{q}}$$

(18) $$W_{d_{a}}=U_{avg}^{d_{a}}⊕U_{max}^{d_{a}}$$

### 3.3. Feature Fusion Layer

After global and local feature extraction, the semantic features $E_{1}$, $E_{2}$ and $E_{3}$ obtain $T_{1}$, $T_{2}$ and $T_{3}$, respectively, through multiple semantic feature channels. $T_{1}$, $T_{2}$ and $T_{3}$ are vector-spliced to obtain the final feature representation Q. The process of the vector splicing is shown in Formula (19).

(19) $$Q=T_{1}⊕T_{2}⊕T_{3}$$

### 3.4. Text Matching Layer

**Figure 6:**
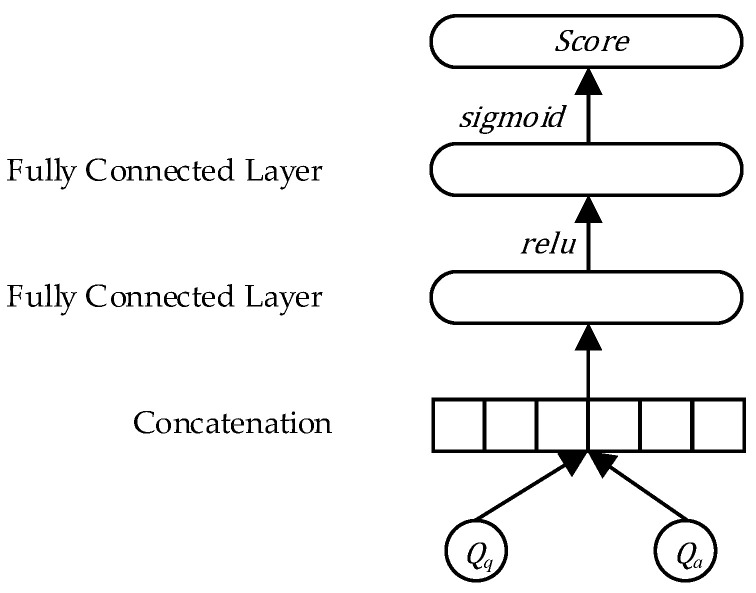
Schematic of text matching layer.

For a given question and candidate answer, whether the candidate answer can answer the question can be judged by calculating the similarity between the representation vector $Q_{q}$ of question q and the representation vector $Q_{a}$ of the candidate answer a and the probability score. In this paper, the fully connected network is used to calculate the similarity between $Q_{q}$ and $Q_{a}$, and the overall flow chart is shown in Figure 6. First, $Q_{q}$ and $Q_{a}$ are vector-spliced, and then passed through multiple fully connected layers to obtain the final matching score. The similarity calculation formula is as follows.

(20) $$score\left(q,a\right)=sigmoid\left(FC\left(Q_{q}^{T}Q_{a}\right)\right)\;$$

Among them, $FC\left(\cdot \right)$ represents the fully connected layer, its neuron activation function is relu, the formula of $sigmoid\left(\cdot \right)$ is $sigmoid\left(x\right)=\frac{1}{1+e^{-x}}$, and the result is a probability score with a value range of $\left[0,1\right]$.

## 4. Experiments and Result Analysis

### 4.1. Experimental Data

The experimental data refer to [33], which was compiled by Feng et al. from foreign insurance websites “https://insurancelibrary.org/ (accessed on 24 May 2016)”. This corpus contains questions and answers based on real scenes in the insurance field, and all the questions raised by users are answered by experts with deep knowledge in the insurance field, so it is a question-and-answer dataset with high credibility. The data in this paper are the translated Chinese version. Before the experiment began, we first carried out data preprocessing, which mainly included four parts: data cleaning, word segmentation, text pinyin conversion and data labeling. In the stage of data cleaning, we removed meaningless characters such as “\u200b” and “&lt” in the insurance corpus by regular expressions, and then loaded a stoplist to delete some meaningless but frequent stop words in the text. In the stage of word segmentation, we used the jieba word segmentation tool to segment words, and loaded the Sogou insurance vocabulary dictionary to identify professional nouns in the insurance corpus. In the stage of text pinyin conversion, we used the pypinyin tool to convert Chinese characters into pinyin, then divided pinyin, and used Word2Vec to train pinyin characters. In the data tagging stage, based on the conventional part-of-speech tagging of jieba, we also defined the part-of-speech for proper nouns in the insurance corpus, which is convenient for model learning-related features.

In this study, according to the ratio of 8:1:1, the insurance corpus data were randomly divided into three parts: a training set, a verification set and a test set, which were used to learn text features, adjust model parameters and evaluate the matching performance of the model, respectively. The overall dataset is shown in Table 1.

There are 185,779 question-and-answer pairs in the insurance question-and-answer corpus, among which the training set contains 12,889 questions and 141,779 data, the verification set contains 2000 questions and 22,000 data, and the test set contains 2000 questions and 22,000 data. Each question includes one positive example and ten negative examples. Negative examples of answers are built from the index of the question. Negative examples are related to the question but are not the correct answer. Part of the experimental data samples after processing is shown in Table 2, where q is the question, a is the answer, and label is the label data. If the label value is 1, it indicates that q and a match; otherwise, if the label value is 0, it indicates that q and a do not match.

### 4.2. Evaluation Index

In this study, Accuracy (Acc), Recall (R) and F1 were used as evaluation indexes to measure the performance of the model. The calculation formula is shown below:

(21) $$Acc=\frac{TP+TN}{TP+TN+FN+FP}$$

(22) $$R=\frac{TP}{TP+FN}$$

(23) $$F1=\frac{2\times P\times R}{P+R}$$

where P is precision, and TP, TN, FP and FN represent as follows:

TP: predicted as Class a, actually as class a, predicted correctly;

TN: the prediction is not Class a, the actual is not Class a, the prediction is correct;

FP: the prediction is Class a, but the actual is not Class a, and the prediction is wrong;

FN: the prediction is not Class a, but the actual result is Class a. The prediction is wrong.

### 4.3. Parameter Setting

The configuration of the experimental environment used in this study is shown in Table 3.

In the experiment, the learning rate was set to 1 × 10^−5^, the number of hidden layers of ERNIE was set to 768, and the number of hidden layers of LSTM was set to 256. The convolution nuclei with the size of {3,4,5} were respectively used for convolution. Each region had 64 convolution nuclei, the epoch was set to 10, and the dropout was set to 0.1.

### 4.4. Contrast Experiment and Results Analysis

In order to verify the performance of the model proposed in this paper on insurance corpus, we selected the following six models for performance comparison:

(1) QACNN [33]: using CNN to learn the distributed vector representation of questions and answers, and using cosine similarity to measure whether they match;
(2) QALSTM [6]: using the BiLSTM to obtain the distributed vector representations of questions and candidate answers, which are weighted based on the correlation between questions and answers, and cosine similarity is used to measure whether they match;
(3) BERT [29]: using the BERT model to generate the context embedding vector of the text, and finally measure whether it matches by cosine similarity;
(4) ERNIE [28]: using the ERNIE model to obtain the context embedding vector of the text, and finally measure whether it matches by cosine similarity;
(5) DARCNN [12]: combining BiLSTM, attention mechanism and CNN, the interaction between questions and candidate answers is established, and multi-dimensional semantic modeling is carried out. Finally, a multi-layer perceptron is used to predict the matching score;
(6) KAAS [16]: obtain the professional information of the vertical field through an external knowledge map, encode the text with word2vec, then obtain the characteristic matrix of questions and answers with BiLSTM, and finally obtain the similarity score by calculating cosine similarity.

The experimental results are shown in Table 4.

As can be seen from Table 4, the Accuracy, Recall and F1 value of the MFBT model proposed in this paper were 78.34%, 78.90% and 78.72%, respectively, which are improved compared with the baseline model. At present, some advanced text-matching models also perform well, but the MFBT model with more text features can still be partially improved. By comparing the experimental results, it can be seen that the QACNN model performed worst in all indicators. Comparing QACNN, QALSTM and DARCNN, the performance of the DARCNN model of LSTM was greatly improved by combining CNN, which shows that the performance of the model can be improved by combining CNN with LSTM. At present, the popular pre-training language model also performs well in text matching. Compared with BERT, ERNIE has improved the accuracy by 2.15%, which is because ERNIE makes up for BERT’s lack of prior knowledge. The KAAS model also introduces an external knowledge graph to expand semantic knowledge after constructing the feature representation of text, so it also performs well in model performance. However, the overall performance of the MFBT model proposed in this paper is obviously superior to the above model, which shows that the matching performance of the model in the insurance field can be effectively improved by fully considering the characteristics of the text and enhancing the semantic representation of the text through multi-feature fusion.

### 4.5. Ablation Experiment and Result Analysis

In order to verify the role of the main modules, we designed several groups of ablation experiments to evaluate the performance of the model. In the ablation experiments, one module was eliminated on the premise that other experimental conditions were consistent, so as to explore the role of each module in the model on the performance of the model. The accuracy rate was used for evaluation. The specific experimental design scheme is as follows:

Method 1: To explore the influence of the word coding mode on model performance, BERT was used for word vector embedding, and the rest of the model remained unchanged.

Method 2: To explore the influence of pinyin character features on the model performance, the pinyin character features were removed from the model, and the rest of the model was kept unchanged;

Method 3: To explore the impact of part-of-speech features on model performance, part-of-speech features were removed from the model, and the rest of the model was kept unchanged;

Method 4: To explore the influence of local semantic features on model performance, TextCNN was removed from the model, and the rest of the model was kept unchanged;

Method 5: To explore the influence of global semantic features on model performance, BiLSTM was removed from the model, and the rest of the model was kept unchanged;

Method 6: To explore the influence of pinyin character features and part-of-speech features on the performance of the model, pinyin features and part-of-speech features were removed from the model, and the rest of the model was kept unchanged.

The experimental results are shown in Table 5. It can be seen that the semantic features in the model had the greatest influence on the performance of the model. Compared with Method 4, the accuracy was improved by 10.3%, and compared with Method 5, the accuracy was improved by 8.16%. This shows that semantics plays an important role in the matching effect of text matching, and the matching accuracy of the model can be greatly improved by deeply mining the semantic features of the text. In addition, enriching the feature input of the model by extracting word features, pinyin character features and part-of-speech features can also improve the performance of the model.

Specifically, BERT trains the model on the basis of word granularity and ignores some proper nouns, thus reducing the accuracy of the model prediction. In this paper, ERNIE is used to extract word features. Since ERNIE introduces external prior knowledge, to some extent, it makes up for the shortcomings of ignoring entity relations and proper nouns when BERT is used for word embedding. Therefore, the text information contained in the insurance corpus is better preserved, and the matching performance of the model is improved. Although the semantic expansion by adding pinyin character features has improved the model performance, compared with other parts, the improvement is the least obvious, at only 0.56%. The reason is that polyphones do not account for a large proportion of the insurance corpus, so there is little room for improvement. Additionally, the addition of part-of-speech features improved the model’s performance by about 1%, indicating that part-of-speech is also helpful for the model to learn the features of the text. By marking special keywords such as proper nouns in the insurance corpus separately, the model can better extract the features of the text, thus improving the model’s matching performance. By comparing the results of Method 6 with those of Method 2, Method 3 and MFBT, it can be seen that the matching performance of the model can be improved by adding pinyin character features and part-of-speech features as a supplement to semantic features on the basis of word features, and the accuracy rate was increased by 1.36% compared with MFBT. Compared with the method of semantic extension to improve the text-matching performance, considering the global and local semantic feature extraction is significant for the improvement of the model. By using BiLSTM to extract the global information of the text and fully considering the role of the context information of the text on the semantic representation, the model performance was improved by 8.2%. TextCNN was used to extract the local information of text, and neural networks with different convolution kernel sizes were used to obtain the semantic features of text at different levels. The performance of the model was improved by 10.3%. It can be seen that the performance of the convolutional neural network was slightly better than that of the cyclic neural network in terms of model performance improvement.

### 4.6. Question-and-Answer Results Analysis

This section makes a visual analysis of the results of the answer selection. Selecting “What is the surcharge for medical insurance levy?” as the question input of the test sample, we can see that its key information includes “charge” and “medical insurance”. After calculation and sorting, the first three candidate answers are as follows.

Candidate answer 1: If you are a high-income senior, you have to pay a surcharge in addition to your MedicareB Part B premium. The fine you pay depends on your income two years ago. In 2013, the total premium of your Part B may range from $147 to $386 per month.

Candidate answer 2: Surcharge on medical insurance is that Australians earn a certain amount of tax, and there is no private medical insurance hospital fee. This surcharge provides health insurance for you and your family, including your spouse, any child under the age of 21 or any student under the age of 25. Surcharge is calculated based on your income of 1 to 1.5 in addition to the 1.5 medical insurance levy paid by most Australian taxpayers.

Candidate answer 3: Medical insurance will assess higher insurance premium, surcharge or levy under several different circumstances. MedicareB Part B insurance premium can be taxed according to your income. Congress decides that people with higher income should cover more surcharges for medical insurance, which is based on the income tax return two years ago. If you fail to register for Part B in time, you may be punished for delaying admission.

From the above matching results, it can be seen that the keywords of the first three recommended candidate answers all include “medical insurance” and all mention “recharge”, which are related to the test questions, and can be used as the answers to the test questions after verification, which has high reference value.

## 5. Discussion

The question-and-answering community based on semantic retrieval has been a research hotspot in the field of artificial intelligence in recent years. The common applications are intelligent chat systems and daily inquiry systems, but there is relatively little research on knowledge question-and-answering in the insurance field. In the text of insurance question-and-answer pairs, the length of answers is generally long, so how to extract effective information from complex sentences brings great challenges. Aiming at the question-and-answer matching task in the insurance question-and-answer community, we mainly completed two aspects of work to improve it. On the one hand, we enriched the semantic representation of the text by extracting various features, including word features, pinyin character features and part-of-speech features. On the other hand, we processed the sequence data by integrating BiLSTM and TextCNN depth models to complete the global and local feature extraction of the text.

Although the multi-feature text matching model proposed in this paper has a certain improvement effect compared with the benchmark model, there are still some improvements to be made. In this study, model verification was only carried out on the dataset of the insurance field, and other professional data fields will be considered later, so as to prove that the model proposed in this paper has certain portability.

## 6. Conclusions

Due to the characteristics of many proper nouns, nonstandard sentences and sparse features in the insurance corpus, the retrieval accuracy of the question-and-answer community in the insurance field is not high. This paper proposes the MFBT text matching model, which extracts word features, pinyin character features and part-of-speech features of the text as the semantic expansion of the text to enhance the semantic feature representation of the text. At the same time, this paper also uses BiLSTM to capture long-distance dependence information and the strong local feature extraction ability of the TextCNN, fully considers the global and local features of the text, and preserves the corpus features to the greatest extent. The text feature representations are input into the text matching layer after vector splicing, thus realizing the semantic matching task of the text. The main contributions of this paper are as follows:

(1) The use of ERNIE for word feature extraction makes up for the lack of prior knowledge in the text feature representation of BERT and other models to a certain extent, and preserves the professional terms of the text in the field of insurance to the greatest extent;
(2) Using pinyin character features as another semantic extension of text can solve the problem of different homophones in the text;
(3) Through the part-of-speech features of the text, the influence of some keywords in the insurance corpus on the model performance is fully considered, so as to improve the feature representation ability of the model;
(4) By combining BiLSTM with TextCNN, our method can comprehensively obtain the context information and local semantic information of the text, thus better representing the text and helping the machine to understand the semantics.

The experimental results of the insurance corpus dataset on the insurance library website show that the model proposed in this paper can perform text representation better and improve text-matching accuracy in the insurance field.

## Figures and Tables

**Table 1:** Overall situation of insurance corpus.

	Training Set	Verification Set	Test Set
Questions	12,889	2000	2000
Correct answers	12,889	2000	2000
Wrong answers	128,890	20,000	20,000
Maximum length of questions	42	31	33
Mean length of problems	5	5	5
Maximum length of answers	878	878	878

**Table 2:** Sample experimental data.

q	a	Label
What does car insurance cover	It depends on the coverage you have. If you are comprehensive...	1
What does car insurance cover	Direct auto insurance is not a kind of auto insurance but buying...	0
What does car insurance cover	In the use of credit scoring and multivariate rating formulas appear...	0
What does car insurance cover	Personal car policy PAP usually includes premiums that include...	0
What does car insurance cover	There was less for a while but in many cases cars...	0
…	…	…
What does car insurance cover	If you asked to cover a stolen car, answer...	0
What does long-term health insurance cover	Good long-term care policies will cover home health care...	1
What does long-term health insurance cover	It’s hard to say how many people have...	0
…	…	…
Whether the home insurance covers the apartment fee	Apartment owners have a special form of homeowner’s insurance that mostly...	0

**Table 3:** Experimental environment.

Development Environment	Parameter
CPU	Intel(R)Core(TM)i7-11700F@2.5 GHz
Video card	NVIDIA GeForce RTX3060 Ti
Operating system	Ubuntu20
Development tool	Pycharm
Programming language	Python3.6
Development framework	Pytorch1.1

**Table 4:** Comparison of the baseline model.

Model	Acc	R	F1
QACNN	68.76%	70.24%	69.48%
QALSTM	70.52%	71.56%	71.03%
BERT	73.54%	73.23%	72.80%
ERNIE	75.69%	75.69%	75.50%
DARCNN	76.21%	76.43%	76.42%
KAAS	77.82%	77.80%	77.81%
MFBT	78.34%	78.90%	78.72%

**Table 5:** Results of the ablation experiments.

Model	Acc	R	F1
Method 1	77.27%	77.27%	77.27%
Method 2	77.78%	77.75%	77.76%
Method 3	77.40%	77.40%	77.40%
Method 4	68.04%	68.12%	68.08%
Method 5	70.14%	70.06%	70.10%
Method 6	76.98%	77.52%	77.25%
MFBT	78.34%	78.90%	78.72%

## Data Availability

Not applicable.
